# Dangerous Effects from the Use of the Tobacco Enema

**Published:** 1846-03

**Authors:** 


*Dangerous Effects from the use of the Tobacco Enema.*—M. Bertini relates in the *Giornale delle Scienze Mediche della Societa Medico-Chirurgica di Torino*, the case of a child, four and a half years old, to whom its parents administered an enema prepared with part of a cigar, in the belief that the child was labouring under worms. When seen by M. Bertini, there was extreme agitation, convulsions, face pale and contracted, eyes fixed, expression of the countenance that of stupefaction, pulse small and weak, cold sweat all over the body, nausea, and the epigastric region painful on pressure. A purgative enema was immediately exhibited, and coffee without sugar given by the mouth. Friction was practised roughly all over the body; friction with sulphuric ether on the temples and epigastrium, and cold affusion on the face. The little patient soon began to amend, and, as soon as deglutition was possible, lemonade was exhibited freely. Two days afterwards the patient was quite restored.—*Ibid.*

